# The Center Problem for the Lotka Reactions with Generalized Mass-Action Kinetics

**DOI:** 10.1007/s12346-017-0243-2

**Published:** 2017-06-13

**Authors:** Balázs Boros, Josef Hofbauer, Stefan Müller, Georg Regensburger

**Affiliations:** 1 Faculty of Mathematics, University of Vienna, Oskar-Morgenstern-Platz 1, 1090 Vienna, Austria; 2 Radon Institute for Computational and Applied Mathematics, Austrian Academy of Sciences, Altenbergerstrasse 69, 4040 Linz, Austria; 3 Johannes Kepler University Linz, Altenbergerstrasse 69, 4040 Linz, Austria

**Keywords:** Chemical reaction network, Power-law kinetics, Center-focus problem, Focal value, First integral, Reversible system, 34C25, 34C07, 92E20

## Abstract

Chemical reaction networks with generalized mass-action kinetics lead to power-law dynamical systems. As a simple example, we consider the Lotka reactions and the resulting planar ODE. We characterize the parameters (positive coefficients and real exponents) for which the unique positive equilibrium is a center.

## Introduction

Lotka [7] considered a series of three chemical reactions, transforming a substrate into a product via two intermediates, ${\mathsf{X}}$ and ${\mathsf{Y}}$. If the reactions producing ${\mathsf{X}}$ and ${\mathsf{Y}}$, respectively, are assumed to be autocatalytic, then the resulting ODE is the classical Lotka–Volterra predator-prey system [8, 9].

Farkas and Noszticzius [4] and Dancsó et al. [2] considered generalized Lotka–Volterra schemes, arising from the Lotka reactions with power-law kinetics. They studied the ODE

1 $$\begin{aligned} \dot{x}&= k_1 \, x^{\hat{p}} - k_2 \, x^{p} y^{q} , \nonumber \\ \dot{y}&= k_3 \, x^{p} y^{q} - k_4 \, y^{\hat{q}} \end{aligned}$$

with positive coefficients $k_1,k_2,k_3,k_4>0$ and real exponents $p,q,\hat{p},\hat{q}\ge 1$. (The special case $p=q=\hat{p}=\hat{q}=1$ is the classical Lotka–Volterra system.) Dancsó et al. [2] provided a local stability and bifurcation analysis. In particular, by finding first integrals, they determined four cases where the ODE admits a center.

In this work, we allow arbitrary real exponents $p,q,\hat{p},\hat{q}\in {{\mathbb R}}$ in the ODE (1) and study the dynamics on the positive quadrant. In addition to the four known cases, we identify two new cases of centers, by showing that they correspond to reversible systems. Moreover, we prove that centers are characterized by these six cases. In a complementary work [1], we provide a global stability analysis for the Lotka reactions with generalized mass-action kinetics.

The paper is organized as follows. In Sect. 2, we elaborate on the chemical motivation of the ODE under study, and in Sect. 3, we present our main result.

## The Lotka Reactions with Generalized Mass-Action Kinetics

As in the original work by Lotka [7], we start by considering a series of net reactions, ${\mathsf{S}}\rightarrow {\mathsf{X}}$, ${\mathsf{X}}\rightarrow {\mathsf{Y}}$, and ${\mathsf{Y}}\rightarrow {\mathsf{P}}$, which transform a substrate into a product. We are interested in the dynamics of ${\mathsf{X}}$ and ${\mathsf{Y}}$ only, in particular, we assume that the substrate is present in constant amount and that the product does not affect the dynamics. As a consequence, we omit substrate and product from consideration and arrive at the simplified reactions

$$\begin{aligned} {\mathsf{0}}\rightarrow {\mathsf{X}}, \, {\mathsf{X}}\rightarrow {\mathsf{Y}}, \, {\mathsf{Y}}\rightarrow {\mathsf{0}}. \end{aligned}$$

To obtain a classical Lotka–Volterra system as in [8, 9], one assumes the first and the second reaction to be autocatalytic, in particular, one defines the kinetics of the reactions as $v_{{\mathsf{0}}\rightarrow {\mathsf{X}}} = k_{{\mathsf{0}}\rightarrow {\mathsf{X}}} [{\mathsf{X}}]$, $v_{{\mathsf{X}}\rightarrow {\mathsf{Y}}} = k_{{\mathsf{X}}\rightarrow {\mathsf{Y}}} [{\mathsf{X}}][{\mathsf{Y}}]$, and $v_{{\mathsf{Y}}\rightarrow {\mathsf{0}}} = k_{{\mathsf{Y}}\rightarrow {\mathsf{0}}} [{\mathsf{Y}}]$ with rate constants $k_{{\mathsf{0}}\rightarrow {\mathsf{X}}}, k_{{\mathsf{X}}\rightarrow {\mathsf{Y}}}, k_{{\mathsf{Y}}\rightarrow {\mathsf{0}}} > 0$ and concentrations $[{\mathsf{X}}],[{\mathsf{Y}}]\ge 0$. In this work, we consider the Lotka reactions with arbitrary power-law kinetics. In terms of chemical reaction network theory, we assume generalized mass-action kinetics [10, 11], that is,

$$\begin{aligned} v_{{\mathsf{0}}\rightarrow {\mathsf{X}}} = k_{{\mathsf{0}}\rightarrow {\mathsf{X}}} [{\mathsf{X}}]^{{{\alpha }}_1} [{\mathsf{Y}}]^{{{\beta }}_1}, \quad v_{{\mathsf{X}}\rightarrow {\mathsf{Y}}} = k_{{\mathsf{X}}\rightarrow {\mathsf{Y}}} [{\mathsf{X}}]^{{{\alpha }}_2} [{\mathsf{Y}}]^{{{\beta }}_2}, \quad v_{{\mathsf{Y}}\rightarrow {\mathsf{0}}} = k_{{\mathsf{Y}}\rightarrow {\mathsf{0}}} [{\mathsf{X}}]^{{{\alpha }}_3} [{\mathsf{Y}}]^{{{\beta }}_3} , \end{aligned}$$

with arbitrary real exponents ${{\alpha }}_1, {{\beta }}_1, {{\alpha }}_2, {{\beta }}_2, {{\alpha }}_3, {{\beta }}_3 \in {{\mathbb R}}$. The resulting ODE for the concentrations $x=[{\mathsf{X}}]$ and $y=[{\mathsf{Y}}]$ amounts to

2 $$\begin{aligned} \dot{x}&= k_1 \, x^{{{\alpha }}_1} y^{{{\beta }}_1} - k_2 \, x^{{{\alpha }}_2} y^{{{\beta }}_2} , \nonumber \\ \dot{y}&= k_3 \, x^{{{\alpha }}_2} y^{{{\beta }}_2} - k_4 \, x^{{{\alpha }}_3} y^{{{\beta }}_3} , \end{aligned}$$

where $k_1=k_{{\mathsf{0}}\rightarrow {\mathsf{X}}}$, $k_2=k_3=k_{{\mathsf{X}}\rightarrow {\mathsf{Y}}}$, and $k_4=k_{{\mathsf{Y}}\rightarrow {\mathsf{0}}}$. Since we allow real exponents, we consider the dynamics on the positive quadrant. In fact, we study an ODE which is orbitally equivalent to (2) on the positive quadrant and has two exponents less,

3 $$\begin{aligned} \dot{x}&= k_1 \, x^{a_1} y^{b_1} - k_2 , \nonumber \\ \dot{y}&= k_3 - k_4 \, x^{a_3} y^{b_3} , \end{aligned}$$

where $a_1={{\alpha }}_1-{{\alpha }}_2$, $b_1={{\beta }}_1-{{\beta }}_2$, $a_3={{\alpha }}_3-{{\alpha }}_2$, $b_3={{\beta }}_3-{{\beta }}_2$. Further, we assume that the ODE admits a positive equilibrium $(x^*,y^*)$ and use the equilibrium to scale the ODE (3). We introduce $K = \frac{k_3}{k_2} \, \frac{x^*}{y^*}>0$ and obtain

4 $$\begin{aligned} \dot{x}&= x^{a_1} y^{b_1} - 1 , \nonumber \\ \dot{y}&= K \left( 1 - x^{a_3} y^{b_3} \right) . \end{aligned}$$

Clearly, the ODE (4) admits the equilibrium (1, 1) which is not necessarily unique, and the Jacobian matrix at (1, 1) is given by

5 $$\begin{aligned} J = \begin{pmatrix} a_1 &{}\quad b_1 \\ -Ka_3 &{}\quad -Kb_3 \end{pmatrix}. \end{aligned}$$

Dancsó et al. [2] studied the ODE (4) in the orbitally equivalent form

6 $$\begin{aligned} \dot{x}&= x^{\hat{p}} - x^{p} y^{q} , \nonumber \\ \dot{y}&= C \left( x^{p} y^{q} - y^{\hat{q}} \right) , \end{aligned}$$

where $\hat{p}=a_1-a_3$, $\hat{q}=b_3-b_1$, $p=-a_3$, $q=-b_1$, and $C=K$. They stated four cases where the equilibrium (1, 1) is a center and provided first integrals. In this work, we identify two new cases and show that they correspond to reversible systems. Moreover, we prove that every center belongs to one of the six cases.

## Main Result

An equilibrium is a *center* if all nearby orbits are closed.

### Theorem 1

The following statements are equivalent.

1. The equilibrium (1, 1) of the ODE (4) with $K>0$ is a center.
2. The eigenvalues of the Jacobian matrix at (1, 1) are purely imaginary, that is, ${{\mathrm{tr}}}\, J=0$ and $\det \,J>0$, and the first two focal values vanish.
3. The parameter values $a_1,b_1,a_3,b_3 \in {{\mathbb R}}$, and $K>0$ belong to one of the six cases in Table 1.

### Proof

1. $\Rightarrow$ 2: If *J* has a zero eigenvalue, that is, $\det \, J=0$, then (1, 1) lies on a curve of equilibria and cannot be a center. Hence, the eigenvalues of *J* are purely imaginary, and all focal values vanish.
2. $\Rightarrow$ 3: For the computation of the first two focal values, $L_1$ and $L_2$, and the case distinction implied by ${{\mathrm{tr}}}\, J=0$, $\det \, J>0$, and $L_1=L_2=0$, see Sect. 3.1.
3. $\Rightarrow$ 1: For the cases (i)–(iv) in Table 1, first integrals have been given by Dancsó et al. [2]. In fact, they determined all the cases for which a first integral can be found by using an integrating factor of the form $x^Ay^B$. See Table 2 and [2, p. 122, Table I].

Case (i) includes the classical Lotka–Volterra systems; the corresponding first integral is of the type of separated variables and was already stated in Farkas and Noszticzius [4]. In case (iv), there is a typo in [2]; the correct formula is $C=\tfrac{1}{\hat{q} - 1}$.

The remaining cases, (r1) and (r2), are reversible systems. See Sect. 3.2.

**Table 1:** Special cases of the ODE (4) with $K>0$ having a center

Case	Parameters			ODE
(i)	$a_1=b_3=0$	$K>0$	$a_3b_1>0$	$$\begin{aligned} \dot{x}&= y^{b_1}-1 \\ \dot{y}&= K(1-x^{a_3}) \end{aligned}$$
(ii)	$$\begin{aligned} a_1&=a_3+1 \\ b_3&=b_1+1 \end{aligned}$$	$K=\textstyle \frac{a_1}{b_3}>0$	$a_1+b_3<1$	$$\begin{aligned} \dot{x}&= x^{a_1}y^{b_3-1}-1 \\ \dot{y}&= \textstyle \frac{a_1}{b_3} (1-x^{a_1-1}y^{b_3}) \end{aligned}$$
(iii)	$$\begin{aligned} a_3&=-1 \\ b_3&=1 \end{aligned}$$	$K=a_1>0$	$a_1+b_1<0$	$$\begin{aligned} \dot{x}&= x^{a_1}y^{b_1}-1 \\ \dot{y}&= \textstyle a_1 (1-\textstyle \frac{y}{x}) \end{aligned}$$
(iv)	$$\begin{aligned} a_1&=1 \\ b_1&=-1 \end{aligned}$$	$K=\textstyle \frac{1}{b_3}>0$	$a_3+b_3<0$	$$\begin{aligned} \dot{x}&= \textstyle \frac{x}{y}-1 \\ \dot{y}&= \textstyle \frac{1}{b_3}(1-x^{a_3}y^{b_3}) \end{aligned}$$
(r1)	$$\begin{aligned} a_1&=b_3 \\ a_3&=b_1 \end{aligned}$$	$K=1$	$|a_1|<|b_1|$	$$\begin{aligned} \dot{x}&= x^{a_1}y^{b_1}-1 \\ \dot{y}&= 1-x^{b_1}y^{a_1} \end{aligned}$$
(r2)	$$\begin{aligned} a_1&=Kb_3 \\ a_3&=Kb_1 \end{aligned}$$	$K=\textstyle \frac{1}{b_3-b_1-1}>0$	$|b_3|<|b_1|$	$$\begin{aligned} \dot{x}&= x^{K b_3}y^{b_1}-1 \\ \dot{y}&= K(1-x^{K b_1}y^{b_3}) \end{aligned}$$

The first four cases were already stated in Dancsó et al [2], where first integrals have been given. The last two cases correspond to reversible systems

**Table 2:** Special cases of the ODE (4) with $K>0$ having a center and the corresponding first integrals and integrating factors (i.f.)

Case	ODE	First integral *V*(*x*, *y*)	i.f. *h*(*x*, *y*)
(i)	$$\begin{aligned} \dot{x}&= y^{b_1}-1 \\ \dot{y}&= K(1-x^{a_3}) \end{aligned}$$	$(x-\frac{1}{a_3+1}x^{a_3+1}) + \frac{1}{K}(y-\frac{1}{b_1+1}y^{b_1+1})$	1
(ii)	$$\begin{aligned} \dot{x}&= x^{a_1}y^{b_3-1}-1 \\ \dot{y}&= \tfrac{a_1}{b_3} (1-x^{a_1-1}y^{b_3}) \end{aligned}$$	$a_1 x+b_3 y - x^{a_1}y^{b_3}$	1
(iii)	$$\begin{aligned} \dot{x}&= x^{a_1}y^{b_1}-1 \\ \dot{y}&= a_1 (1-\tfrac{y}{x}) \end{aligned}$$	$- \frac{a_1}{a_1-1}x^{-a_1+1}-\frac{1}{b_1+1}y^{b_1+1}+x^{-a_1}y$	$x^{-a_1}$
(iv)	$$\begin{aligned} \dot{x}&= \tfrac{x}{y}-1 \\ \dot{y}&= \tfrac{1}{b_3}(1-x^{a_3}y^{b_3}) \end{aligned}$$	$- \frac{1}{a_3+1}x^{a_3+1}-\frac{b_3}{b_3-1}y^{-b_3+1}+xy^{-b_3}$	$y^{-b_3}$
(r1)$\cap$(r2)	$$\begin{aligned} \dot{x}&= x^{b_1+2}y^{b_1}-1 \\ \dot{y}&= 1-x^{b_1}y^{b_1+2} \end{aligned}$$	$(\frac{1}{x}+\frac{1}{y})^{-(b_1+1)} (1 + (xy)^{-(b_1+1)})$	$(x+y)^{-(b_1+2)}$

If ${{\alpha }}$ is zero in $\frac{x^{{\alpha }}}{{{\alpha }}}$ (in a first integral), replace $\frac{x^{{\alpha }}}{{{\alpha }}}$ by $\ln x$

### Case Distinction

Using ${{\mathrm{tr}}}\, J=0$, that is, $a_1=K b_3$ by Eq. (5), we compute $\det \, J$ and the first two focal values, $L_1$ and $L_2$. We find

$$\begin{aligned} \det \, J =K (a_3 b_1 - b_3^2 K) \end{aligned}$$

and note that $\det \, J>0$ implies $a_3,b_1\ne 0$. Further, using the Maple program in [6], we find

$$\begin{aligned} L_1 = \frac{\pi }{8} \, \frac{K b_3 \left[ b_1 (1 + a_3 - a_3 K - b_3 K) - a_3 (1 - b_3) K \right] }{\sqrt{\det \, J} \, b_1} . \end{aligned}$$

Expressions for $L_2$ (in case $L_1=0$) will be given below.

We show that all parameters $a_1,b_1,a_3,b_3 \in {{\mathbb R}}$ and $K>0$ in the ODE (4) for which

$$\begin{aligned}\begin{gathered} {{\mathrm{tr}}}\, J=L_1=L_2=0 \\ \text {and } \det \, J>0 \end{gathered}\end{aligned}$$

belong to one of the six cases in Table 1.

To begin with, $L_1=0$ implies either

- $$b_3=0$$,
- $b_1=\frac{a_3 (1 - b_3) K}{D}$, where $D=1 + a_3 - a_3 K - b_3 K$ and $D\ne 0$, or
- $D=0$ and $b_3 = 1$. In this case, $(1+a_3)(1-K)=0$ and either
  1. $$b_3=1$$, $$a_3=-1$$ or
  2. $$b_3=1$$, $$K=1$$.

In case (a), where $b_3=0$ (and hence $a_1=0$), we find $\det \, J = K a_3 b_1$. Hence, the situation is covered by case (i) in Table 1.

In case (b), where $D \ne 0$, we find $b_3 \ne 1$ (otherwise $b_1=0$) and, using the Maple program in [6],

$$\begin{aligned} L_2 = \frac{\pi }{288} \, \frac{(a_3 + b_3)^2 b_3 (1 + a_3 - b_3 K) (1-b_3 K) (1-K) (1 + a_3 + K - b_3 K)}{\sqrt{\det \, J}D(1-b_3)} . \end{aligned}$$

Now, $L_2=0$ implies that at least one of six factors is zero. The first subcase $a_3+b_3=0$ implies $D=1-b_3$ and hence $b_1=a_3 K$ and $\det \, J = 0$. As shown above, the subcase $b_3=0$ is covered by case (i) in Table 1. The subcase $1 + a_3 - b_3 K=0$ implies $D=-a_3K$ and hence $b_1=b_3-1$. That is, $a_1=a_3+1$, $b_3=b_1+1$, and hence $\det \, J=K(1-a_1-b_3)$ which corresponds to case (ii). The subcase $b_3 K=1$ (and hence $a_1=1$) implies $D=a_3(1-K)$ and hence $b_1=-1$. Now, $\det \, J=-K(a_3+b_3)$, and the situation is covered by case (iv). The subcase $K=1$ (and hence $a_1=b_3$) implies $D=1-b_3$ and hence $b_1=a_3$. Now, $\det \, J=b_1^2-a_1^2$, and the situation is covered by case (r1). Finally, the subcase $1 + a_3 + K - b_3 K=0$ implies $D=-(1+a_3)K$ and hence $b_1=\frac{a_3(1-b_3)}{-(1+a_3)}=\frac{a_3}{K}$. That is, $a_1=Kb_3$, $a_3=Kb_1$ and hence $K=\frac{1}{b_3-b_1-1}$, $\det \, J=K^2(b_1^2-b_3^2)$ which corresponds to case (r2).

In case (c1), where $b_3=1$ and $a_3=-1$ (and hence $a_1=K$), we find $\det \, J=-K(a_1+b_1)$. Hence, the situation is covered by case (iii) in Table 1. In case (c2), where $b_3=1$ and $K=1$, we find

7 $$\begin{aligned} L_2 = \frac{\pi }{288} \, \frac{a_3 (1 + a_3) (1 + b_1) (a_3-b_1)}{\sqrt{\det \, J} \, b_1} . \end{aligned}$$

Now, $L_2=0$ implies that at least one of four factors is zero. The first subcase $a_3=0$ implies $\det \, J<0$. As shown above, the subcase $a_3=-1$ is covered by case (iii). Finally, the subcase $b_1=-1$ is covered by case (iv), and the subcase $a_3=b_1$ is covered by case (r1).

### Reversible Systems

Let $R: {{\mathbb R}}^2 \rightarrow {{\mathbb R}}^2$ be a reflection along a line. A vector field $F :{{\mathbb R}}^2 \rightarrow {{\mathbb R}}^2$ (and the resulting dynamical system) is called reversible w.r.t. *R* if

$$\begin{aligned} F\circ R = - R \circ F . \end{aligned}$$

It is easy to see that, for any function $f :{{\mathbb R}}^2 \rightarrow {{\mathbb R}}$, the system

8 $$\begin{aligned} \dot{x}&= f(x,y) \nonumber \\ \dot{y}&= - f(y,x) \end{aligned}$$

is reversible w.r.t. the reflection $R :(x,y) \mapsto (y,x)$. The following is a well-known fact, see e.g. [12, 4.6571] or, more generally, [3, Theorem 8.1].

*An equilibrium of a reversible system which has purely imaginary eigenvalues and lies on the symmetry line of R is a center.*

Now we are in a position to deal with the last two cases in Table 1.

Case (r1):

$$\begin{aligned} \dot{x}&= x^{a_1}y^{b_1}-1 ,\\ \dot{y}&= 1-x^{b_1}y^{a_1} . \end{aligned}$$

This vector field is of the form (8), and hence it is reversible.

Case (r2):

$$\begin{aligned} \dot{x}&= x^{K b_3}y^{b_1}-1 , \\ \dot{y}&= K(1-x^{K b_1}y^{b_3}) , \end{aligned}$$

where $K = \frac{1}{b_3-b_1-1}$. We apply the coordinate transformation $u = x^K, v = y^{-1}$ and obtain

$$\begin{aligned} \dot{u}&= Kx^{K-1} ( x^{K b_3}y^{b_1}-1) = K u^{1-\frac{1s}{K}} (u^{b_3} v^{-b_1} - 1) , \\ \dot{v}&= - K y^{-2} (1-x^{K b_1}y^{b_3}) =- K v^2 (1-u^{b_1}v^{-b_3}) . \end{aligned}$$

Finally, we multiply the vector field with the positive function $K^{-1} v^{b_1}$ and obtain

$$\begin{aligned} \dot{u}&= u^{1-\frac{1}{K}+ b_3} - u^{1-\frac{1}{K}}v^{b_1} , \\ \dot{v}&= - v^{2 +b_1} +u^{b_1}v^{2+b_1-b_3} . \end{aligned}$$

Since $1-\frac{1}{K} = 2+b_1-b_3$, this vector field is of the form (8), and hence it is reversible.

Since (r1) and (r2) lead to centers, analytic first integrals must exist. However, it seems difficult to find them. So far we succeeded only in the intersection of (r1) and (r2), that is, the case where $a_1 = b_3 = b_1 + 2$, $a_3=b_1$, $K=1$, and $b_1<-1$ (a one parameter family). See Table 2.

### Limit Cycles

Dancsó et al. [2] and Boros et al. [1] identified ODEs (1) and (2) that admit one limit cycle (via a super- or sub-critical Hopf bifurcation). As a simple consequence of our characterization of the center variety, we can construct ODEs (4) with two limit cycles via a degenerate Hopf or Bautin bifurcation, see [5, Sect. 8.3]. We pick a system with ${{\mathrm{tr}}}\, J=L_1=0$ and $L_2 \ne 0$, in particular, we consider case (c2) of our case distinction: we take $b_3 = a_1 = 1$, $K=1$ and hence ${{\mathrm{tr}}}\, J=L_1=0$ and choose $b_1$ and $a_3$ such that $L_2 <0$ with $L_2$ given by Eq. (7); for example, $b_1 = -2$, $a_3 = -3$ or $b_1 = 2, a_3 = 1$. If we now slightly perturb *K* (keeping $a_1=K$) such that $L_1 > 0$ (and ${{\mathrm{tr}}}\, J=0$), the resulting system has a stable limit cycle. Finally, if we slightly change $a_1$ such that ${{\mathrm{tr}}}\, J< 0$, we create a small unstable limit cycle via a subcritical Hopf bifurcation.

It remains open, if the ODE (4) admits more than two limit cycles. For a computational algebra approach to this question, see [13].
